# Bee pollination increases yield quantity and quality of cash crops in Burkina Faso, West Africa

**DOI:** 10.1038/s41598-017-17970-2

**Published:** 2017-12-18

**Authors:** Katharina Stein, Drissa Coulibaly, Kathrin Stenchly, Dethardt Goetze, Stefan Porembski, André Lindner, Souleymane Konaté, Eduard K. Linsenmair

**Affiliations:** 1 Biocenter, Department of Animal Ecology and Tropical Biology, University of Wuerzburg, Wuerzburg, Germany; 2 Unité de Formation et de Recherche des Sciences de la Nature, Unité de Recherche en Ecologie et Biodiversité, Université Nangui Abrogoua Abidjan, Abidjan, Ivory Coast; 3 Faculty of Organic Agricultural Sciences, Organic Plant Production and Agroecosystems Research in the Tropics and Subtropics, Universität Kassel, Kassel, Germany; 4 Institute of Biological Sciences, Department of Botany and Botanical Garden, University of Rostock, Rostock, Germany; 5 Centre for International Postgraduate Studies of Environmental Management–CIPSEM, Technische Universität Dresden, Dresden, Germany

## Abstract

Mutualistic biotic interactions as among flowering plants and their animal pollinators are a key component of biodiversity. Pollination, especially by insects, is a key element in ecosystem functioning, and hence constitutes an ecosystem service of global importance. Not only sexual reproduction of plants is ensured, but also yields are stabilized and genetic variability of crops is maintained, counteracting inbreeding depression and facilitating system resilience. While experiencing rapid environmental change, there is an increased demand for food and income security, especially in sub-Saharan communities, which are highly dependent on small scale agriculture. By combining exclusion experiments, pollinator surveys and field manipulations, this study for the first time quantifies the contribution of bee pollinators to smallholders’ production of the major cash crops, cotton and sesame, in Burkina Faso. Pollination by honeybees and wild bees significantly increased yield quantity and quality on average up to 62%, while exclusion of pollinators caused an average yield gap of 37% in cotton and 59% in sesame. Self-pollination revealed inbreeding depression effects on fruit set and low germination rates in the F1-generation. Our results highlight potential negative consequences of any pollinator decline, provoking risks to agriculture and compromising crop yields in sub-Saharan West Africa.

## Introduction

Insect pollination of flowering plants is a process of significant importance in terrestrial environments, and it provides vital ecosystem services for human well-being^1,2^ such as crop production. About 75% of agricultural crop species rely, to some degree, on animal pollination, and about one-third benefit from cross-pollination by developing higher fruit quantity and/or quality^3–5^. Among other insects, bees are known as one of the most important groups of pollinators worldwide^6,7^; a decrease of this pollination service could potentially reduce yields by ca. 40%^3^. However, non-bee insects have been shown to be important pollinators, too^8^. Land-use change and rapid habitat transformation during recent decades are seen as important drivers of insect pollinator declines^2,9^, thereby increasing the risk of future pollination deficits in areas of high, and increasing, pollination demands^10^. Areas where food production mostly depends on animal pollination are also those from which the fewest data are available^11,12^; this is due to lack of infrastructure and funding in many areas of the world, particularly in developing countries. The same areas are often poorly buffered against disruption of ecosystem service provision from whatever cause, and for this reason, effects of any ecological incidents on human well-being could be more severe here than elsewhere^13^.

Burkina Faso’s economic development largely depends on agriculture, with cotton as main export product (over 60% of the total export volume), making it the main cotton producer in sub-Saharan Africa^14,15^. Sesame ranks third among the top ten export commodities of the country. According to FAO^16^ cotton is sold as lint (265,500 tonnes in 2014) and seed (12,589 tonnes in 2014), sesame is sold as seed (321,837 tonnes production on 506,095 ha area harvested in 2014). Around 80% of the population relies on subsistence farming, as it is true for West Africa in general^17^, with the majority of agricultural production on properties of less than 2 ha up to 6 ha. A standard field of a Burkinabé farmer is of ca. 1 ha in size and a farmer usually cultivates a diversity of cotton, maize, sorghum, millet, sesame, etc. on each 1 ha field^18^.

Cotton and sesame are known to be able to self-pollinate, though the levels of selfing and outcrossing differ in the literature^19,20^. Additionally, only few studies have investigated the quality of the offspring in terms of germination rate, survival and growth performance in comparison to individuals resulting from self-pollination. Furthermore, different crop varieties can exhibit variable levels of pollinator dependence and possible yield deficits, hence an investigation of potential impacts of variety on pollination is important^21,22^.

Despite the variable level and mode of pollination, bees have been reported to be important pollinators of cotton and sesame^23–25^. Some systematic assessments of pollinator species and their relative pollination service contribution to the yield quantity and quality of these two important cash crops have been carried out^26,27^, but estimations of the relative economic benefits of bee pollination have rarely been made. Most studies assessing the contribution of insect pollinators to yield quantity and quality were conducted in Europe and North America^25,28,29^. Studies in Africa mostly concentrate on South African fruit^30,31^ and seed crops^32–34^. Additionally, the majority of studies are based on large-scale farms, while smallholding crop systems with fields smaller than 2 ha have received less attention in ecosystem-service research^35,36^. Following FAO, the insect pollination economic value (IPEV) for West Africa is estimated as 5.6 Billion US Dollars, which is the highest on the African continent along with one of the highest vulnerability rates in terms of pollination (i.e., IPEV/total production economic value)^11,37^.

In order to assess the supply of pollination services provided by insects to upland cotton and sesame in Burkina Faso, this study utilizes a novel combination of pollinator effectiveness assays, pollinator dependence manipulations and quality measures of F1-generation individuals from selfing or outcross-pollination. We have (1) determined the level of self-compatibility and the selfing rate of the crop varieties; (2) determined the pollinator species of cotton and sesame, and quantified their relative effectiveness; (3) quantified the demand for insect pollination services; (4) investigated signs of inbreeding depression after self-pollination; and (5) estimated the economic benefits of pollinators to smallholder production.

## Methods

### Study area and study sites

Field work was conducted from June to November 2015 in the southwest of Burkina Faso. This period covers the rainy season and outset of the dry season, which equally mark the flowering and harvesting season of cotton and sesame in Burkina Faso.

We selected the variety FK37 of conventional upland cotton (*Gossypium hirsutum*, Malvaceae), which is the most commonly used variety in Burkina Faso with more than 50% of all cotton varieties cultivated. In the study region of southwest Burkina Faso, this variety was cultivated on 100% of all cotton fields in 2015 (79% in 2017, depending on seed availability; local cotton producer society, SOFITEX, pers. comm.). For sesame (*Sesamum indicum*, Pedaliaceae), we selected the variety S-42, which is most widely cultivated, and covers over 80% of the cultivated areas in Burkina Faso. Three sites for exclusion experiments and pollinator monitoring in cotton and sesame fields were chosen: Dano (11°08'56.566″ N, 003°03'36.446″ W), Bontioli (10°48'26.393″ N, 003°04'39.564″ W) and Nazinga (11°06'34.998″ N, 001°29'07.181″ W), at elevations between 271 and 448 m a.s.l. All sites are located in the south-Sudanian vegetation zone and are characterized by a mosaic of agricultural land, small villages and fragments of near-natural savanna woodlands. The average proportion land cover (%) of grass-, shrub- and tree savannas of the sites amounts to 72.98% ± 18.15%. Average crop land cover (farmland and fallows) amounts to 16.86% ± 18.59%^38,39^. Smallholder agricultural fields were ca. 1 ha each in size, typically embedded in a matrix of other small fields of wind-pollinated cereal crops such as maize, millet and sorghum, or entomophilous crops such as beans and groundnut, with a maximum distance of 500 m (mean 81 m ± 101 m SD) to the next near natural savanna fragment.

At all sites farmers were requested to continue their usual farming practice during the study period: fertilizers were applied at the beginning of the sowing season, insecticides and fungicides were irregularly applied depending on the infestation rate and financial resources of the farmers. Weeds were removed manually. The management practices at all sites were alike. Only at the study site of Bontioli, a neighboring farmer kept 5 beehives of managed honeybees in the vicinity of about 2 km to our study fields. The beehives were not moved towards the fields.

### Breeding system, seed germination and sapling performance

To investigate the level of self-pollination, self-compatibility and potential signs of inbreeding depression in the studied crop varieties, pollination experiments were conducted following Dafni^40^ (see Supplementary Table S1).

Cotton and sesame both have one-day flowers. All manipulations were carried out on the only day of flower anthesis, which ensured stigma receptivity and thus successful hand pollination experiments. Flowers usually open around 7:00 a.m., and pollen is released 30 to 60 min later. For our experiments, 50 plants of cotton and 50 plants of sesame were randomly selected from one field each. Five different pollination treatments were conducted per individual plant. For each treatment, we used two flowers per plant (i.e. 10 flowers per plant were treated). In total we investigated 100 flowers per treatment. Each individual plant was subjected to all pollination treatments rather than only to one treatment. This approach was made to avoid assessing the impact of the individual plant on reproductive performance rather than the impact of the treatment itself. For bagging of buds and flowers we used perforated pollination bags (hole diameter: 0.5 mm). Emasculation (removal of anthers), if required, was done in the morning between 7:00 and 7:30 a.m. before anthers released pollen. Due to high fragility of sesame flowers and difficult access to the anthers, the treatments hand-crossing (HC), hand-selfing (HS) and spontaneous selfing (SS) were carried out in a greenhouse of the Botanical Garden, University of Rostock, Germany under simulated field conditions (day/night: 32 °C/25 °C, 12 h light, 12 h dark). The open pollination treatments control (C) and natural outcrossing (OC) were conducted with the same seed variety in Burkina Faso since they required the presence of natural pollinators.

When fruits were mature, we recorded whether flowers set fruit, and if so, the number and weight of seeds per fruit. Mean fruit set, overall mean number of seeds per fruit and mean seed weight were then calculated for each treatment. Fruit set is calculated as number of fruits divided by the number of flowers.Two indices related to selfing were calculated: 1) the selfing rate “S”^41^ calculated as:

1 $${\rm{S}}=\frac{Px-Pc}{Px-Ps}$$

where *Px* are seeds resulting from open cross-pollination (OC), *Pc* are seeds resulting from open pollination (C) and *Ps* are seeds resulting from self-pollination (SS), and 2) the index of self-incompatibility “ISI”^42,43^ as the average fruit set by hand self-pollination (HS) divided by the average fruit set by hand cross-pollination (HC).

Inbreeding depression for fruit set, seed production, seed weight, and seed germination were calculated by the inbreeding depression index^44^ as the relative performance of cross types (RP) following the formula:

2 $${\rm{RP}}=1-\frac{Ws}{Wo},\mathrm{if}\,Ws\le Wo$$

where *Ws* is the mean performance of selfed progeny (SS) and *Wo* is the mean performance of out-crossed progeny (OC). This index varies from −1 to 1. Positive values indicate that outcrossed progeny outperform selfed progeny (i.e. selfed progeny suffers from inbreeding depression), negative values indicate the opposite case^44^.

To evaluate seed germination, 270–370 cotton seeds from each pollination treatment (corresponding to the number of available seeds, total number of seeds: 1650) were placed in Petri dishes (10 seeds per dish) on filter paper moistened with purified water and maintained in a climate conditioning chamber at temperatures of 32 °C (day) and 25 °C (night) with artificial lighting and a photoperiod of 12 h from 6:00 a.m.–6:00 p.m.^45^.

Each day, we counted germinated seeds until all seeds had either germinated or decayed.

The same protocol applied to sesame, but with placing the seeds (210–400 sesame seeds from each pollination treatment, total number of seeds: 2249) on substrates (pots) in the greenhouse under similar conditions. All Petri dishes and pots were manually randomized every day (i.e. array in climate chamber and greenhouse, respectively) to avoid impacts of varying microclimatic and light conditions.

After germination, 50 cotton seedlings per treatment were transferred to pots filled with standard substrate (sand-turf mixture) in the greenhouse where the F1-individuals were cultivated. For sesame, seedlings remained in their pots where they already had germinated. After 35 days of cultivation, height and number of leaves per sapling were measured.

### Pollinator species and their effectiveness

To assess pollinating species and their ability to pollinate cotton and sesame flowers, we determined the flower-visiting species and their pollination effectiveness in terms of fruit set and fruit quality. At all three study sites, 100 flowers each of cotton and sesame in bud stage were bagged with pollination bags and marked. In total we assessed flower visitors and their effectiveness as pollinators on 300 flowers of cotton and 300 flowers of sesame. Every day at 7:00 a.m., we checked if flowers had opened. If open, the bag was removed and we waited at a slight distance for the first pollinator to enter the focal flower. When leaving the flower, the pollinator was caught, etherized and stored in 70% ethanol. Thereon, the flower was bagged again after the insect visit. Care was taken that weather conditions were similar (sunny to bright, no rain) on all sampling days. Each pollinator specimen was numbered according to the visited flower and then pinned and identified to genus or species (voucher specimens are held at the Royal Belgian Institute of Natural Sciences, Brussels). At the end of the season, we recorded whether each visited flower set fruit, and if so the quality parameters such as the number of intact and non-intact seeds, seed weight, and additionally for cotton, fiber weight (lint weight) per fruit. For each visitor species, we calculated mean values and standard deviations for all parameters.

### Pollinator dependence

To investigate to which extent the focal cash crop species benefit from insect-mediated outcross pollination in comparison to simulated total absence of pollinators, pollinator exclusion experiments were conducted on 11 fields of cotton and sesame each: 4 fields of cotton and sesame each were selected at a maximum distance of 1 km to the nearest semi-natural savanna habitat in “Nazinga” and “Bontioli”. However, at “Dano”, 3 fields per crop species were chosen due to infrastructural restrictions.

On each field, 25 plants were selected randomly. Three pollination treatments were conducted per plant: (1) natural open pollination, OPEN; (2) spontaneous self-pollination, SELF, and (3) natural outcross-pollination, CROSS (see Supplementary Table S1). The natural open pollination (control) includes self- and cross-pollination. Due to the selfing ability of both crop species, we added the outcrossing treatment to investigate the pure contribution of insect-mediated pollen transfer from other conspecifics (cross-pollination). Each pollination treatment was applied to two flowers per plant. In total we had 275 plants of cotton and 275 plants of sesame (100 plants in Nazinga and 100 plants in Bontioli due to 25 plants on 4 fields, and 75 plants in Dano due to 25 plants on 3 fields). Since we used two flowers per plant, the experiments were executed with a total of 550 flowers per crop and treatment.

Cotton plants usually produce only one open flower per day. In rare cases, where two flowers were open on the same day, one of the flowers was bagged and used for an exclusion treatment to prevent self-pollination that occurs from pollinator movement between flowers on the same plant (geitonogamy). In sesame, inflorescences appear to bare several open flowers, most of which are old flowers from the day before, and not being receptive to pollen anymore. We only used flowers that were produced during the peak of the flowering season from mid-July until mid-August (i.e. not the very first and the very last flowers produced by a plant), when the plants had reached their full height, to avoid impacts of potential resource limitation on fruit set in early growth and late fruit ripening periods. At the end of the season, we recorded whether each treated flower set fruit, and the quality parameters: 1) number of intact seeds, 2) number of non-intact seeds, 3) seed weight, additionally 4a) fiber weight (lint weight) per fruit for cotton and 4b) fruit weight for sesame.

### Economic valuation of pollination – yield gap

Economic benefits of pollination services to producers were calculated for each crop by comparing the fruit quantity from open natural pollination (mixed self- and cross-pollination), outcross-pollination and pollinator-exclusion treatments (spontaneous self-pollination)^46^. Cotton yield is determined as the weight of cotton fibers (lint weight) and seeds together, with the pericarp removed. Hence, we summed up fiber and seed weight recorded for each fruit of each treatment from the pollinator exclusion experiments, calculated the mean values and the difference of yield resulting from outcrossing and selfing to the natural open pollination (control). In sesame, only the seeds are sold. Hence, we used the seed weight data for economic valuation. Yield gap, defined here as the difference in crop yield between natural open pollination and self-pollination (pollinator exclusion), was calculated as kg*ha^−1^ based on average total yield quantity of the study fields (data obtained from the local cotton producer society SOFITEX and the local sesame farmer community). Based on the market prices in Burkina Faso in 2015, yield gaps and economic benefits through insect pollination were valued by multiplying the yield quantity [kg*ha^−1^] by the lowest national market price per kg (cotton: 0.38 USD or 225 Francs CFA, sesame: 0.43 USD or 250 Francs CFA)^47^ to state realistic values. The farmers´ expenses for seeds, fertilizers and pesticides per hectare (cotton: 140.87 USD or 82875 Francs CFA, sesame: 34.00 USD or 20000 Francs CFA)^47^ were subtracted. Human labor was not monetarily considered in this calculation.

### Data analysis

We fitted generalized linear mixed models (GLMER) to analyse the effect of pollination treatment on fruit set with a binomial data distribution (fruit set = 1, no fruit set = 0). To analyse and compare the effect of pollination treatment on fruit quality parameters of cotton and sesame, we used linear mixed-effects models (LMER) considering in both models field and plant as random grouping factors. Multiple comparisons were conducted using Tukey procedure with *P*-value ≤ 0.05 as threshold for significance. All quantity and quality parameter data refer to field measures at site in Burkina Faso. Only seed germination of sesame seeds deriving from self-pollination refers to the seeds that were produced in greenhouses in Germany. Differences in group means of seed germination after treatment (manually randomized during data collection) were analysed using Kruskall-Wallis One-Way-ANOVA on Ranks due to non-normal distribution of data and pairwise multiple comparison procedures (Tukey Test or Dunn´s respectively) with *P*-value of ≤ 0.05 as the threshold of significance. Data of the follow-up seedling performance experiment were analysed using LMER with Petri dish (cotton) or pot (sesame) as random grouping factor. Linear mixed-effects models were further used to calculate pollinator effectiveness by separating four bee species that visited cotton flowers most frequently from wasps (exclusively Scoliidae species) and from remaining visitors. The dataset of the latter was pooled due to low visitor numbers (less than ten visits per species). The effect of single pollinator species on fruit set was analysed using GLMER and quality parameters, namely seed weight, fibre weight and number of non-intact seeds, were analysed using LMER with field as random grouping factor. For sesame, no statistical test regarding pollinator efficiency was feasible due to inhomogeneity of visitor numbers. Statistical analyses and in part figure production were conducted in R version 3.3.0^48^ with additional functions provided by the R packages MASS^49^, lme4^50^, lmerTest^51^ and multcomp^52^.

### Data availability Statement

The datasets generated during and/or analysed during the current study are available from the corresponding author on reasonable request.

## Results

### Breeding system, seed germination and sapling performance

In both cotton and sesame, pollination treatments revealed a mixed mating system. The cotton variety used in this study has a high ability to set fruit without pollinators via spontaneous selfing (61%), whereas this ability was lower in sesame (20%). The selfing rate ‘S’ accounts for 0.143 in cotton and 0.016 in sesame (i.e. ca. 14% of cotton seed production and ca. 2% of sesame seed production per fruit derive from self-pollination). The hand pollination experiments revealed an index of self-compatibility (ISI) of 0.91 in cotton and 0.71 in sesame. Hence, both crops are partially self-compatible (ISI of 1 = fully self-compatible).

The relative performance of cross types RP (inbreeding depression index) in cotton was determined as 0.197 for fruit set, 0.259 for seed production, 0.206 for seed weight and 0.347 for seed germination rate. In sesame, RP amounted to 0.369 for fruit set, 0.45 for seed production, 0.71 for seed weight and 0.97 for seed germination rate (positive values from 0 to 1 indicate inbreeding depression of selfed progeny, negative values to −1 would indicate outbreeding depression). The germination rate of control seeds was 61.35% in cotton and 26.29% in sesame (C vs. OC, cotton: ANOVA, F_(4,160)_ = 0.536, ns.; sesame: Q = 4.753, *P* < 0.05), whereas the germination rate of seeds resulting from outcross-pollination was highest in both crops (cotton: 72.16%, sesame: 62.16%). Seeds resulting from spontaneous self-pollination germinated least (cotton: 47.09%, sesame: 1.81%; OC vs. SS: cotton: ANOVA, F_(4,160)_ = 0.643, *P* < 0.001; sesame: Q = 7.918, *P* < 0.05).

In cotton, the saplings of the F1-generation resulting from self-pollination showed a trend towards reduced height and number of leaves in comparison to outcrossed saplings (OC vs. SS: height: LMER, *t*_26_ = −1.8, *P* = 0.08; number of leaves: LMER, *t*_23_ = −1.7, *P* = 0.09). Whereas outcrossed saplings had a mean height of 33.20 cm (SD = 12.49) and mean number of leaves of 5.89 (SD = 2.08), saplings from self-pollination were smaller in height (mean: 23.93, SD = 17.76) and had fewer leaves (mean: 4.47, SD = 3.09).

In sesame, no significant difference in the performance of the offspring was found between self-pollination and outcrossing treatments.

### Pollinator species and their effectiveness

The diversity of flower visitor species differed between cotton and sesame. We recorded the semi-wild (few colonies are managed, while the majority is feral) honeybee *Apis mellifera*, twenty-six wild bee species and four species of wasps as flower visitors in cotton. Six wild bee species did not initiate fruit set; all other species are considered as pollinators. Flowers of sesame were visited by *Apis mellifera* and four wild bee species, but only the honeybees and two wild bee species initiated fruit set and are thus considered as pollinators. No other animal taxa or insects were observed visiting the cotton and sesame flowers during the effectiveness experiments (Table 1).

**Table 1:** Taxonomic list of Hymenoptera visitor species to the flowers of cotton (*Gossypium hirsutum*, Malvaceae) and sesame (*Sesamum indicum*, Pedaliaceae), their number (n) of visits and their ability to initiate fruit set. Crosses indicate the initiation of fruit set, dashes no successful pollination. Flower visitors were recorded on 300 flowers per crop species, at three sites in the south of Burkina Faso, West-Africa during the flowering season in summer 2015. Scoliidae are scoliid wasps; Apidae are long-tongued bees including honeybees, small and large carpenter bees and stingless bees; Halictidae are sweat bees and Megachilidae mason bees.

Family	Flower visitor species	n visits	Initiating fruit set
	**Cotton**		
Apidae	*Amegilla acraensis*	6	x
	*Amegilla nubica*	1	x
	*Amegilla torrida*	1	—
	*Apis mellifera*	150	x
	*Braunsapis* sp.1	2	x
	*Braunsapis* sp.2	1	x
	*Braunsapis* sp.3	9	x
	*Braunsapis* sp.4	1	x
	*Ceratina* sp.1	15	x
	*Ceratina* sp.2	4	x
	*Ceratina* sp.3	2	x
	*Hypotrigona gribodoi*	11	x
	*Hypotrigona squamuligera*	1	x
	*Tetralonia fraterna*	59	x
Halictidae	*Austranomia* sp.1	3	x
	*Acunomia ivoiriensis*	1	—
	*Lasioglossum atricrus*	1	—
	*Lasioglossum nairobiensis*	3	x
	*Lasioglossum transvaalensis*	2	x
	*Lasioglossum* sp.4	1	x
	*Leuconomia bouyssoui*	1	x
	*Leuconomia granulata*	3	-
	*Macronomia armatula*	1	x
	*Steganomus junodi*	1	x
	*Trinomia orientalis*	1	—
	*Trinomia cirrita*	1	—
Megachilidae Scoliidae	*Megachile eurynero*	1	x
	*Campsomerella pseudocollaris*	5	x
	*Cathimeris hymenaea*	4	x
	*Micromeriella hyalina*	2	x
	*Lacosia lateralis*	5	x
	**Sesame**		
Apidae	*Amegilla* sp.2	1	—
	*Apis mellifera*	290	x
	*Hypotrigona gribodoi*	2	x
	*Xylocopa ustulata*	2	—
Megachilidae	*Chalicodoma mephistolica*	5	x

The effectiveness assays revealed that not all pollinators were equally effective, and the relative abundance of different pollinator species varied strongly. In cotton, the most abundant pollinator species was the honeybee *Apis mellifera* with 150 visits followed by the native wild bee species *Tetralonia fraterna* (long-horned bee; 59 visits), the small carpenter bee *Ceratina* spec.1 (15 visits) and the stingless bee *Hypotrigona gribodoi* (11 visits). All other twenty-three wild bee species and four Scoliid wasp species were infrequent flower-visitors (Table 1). Only pollination by honeybees significantly increased fruit set, whereas all other pollinator species had no significant effect on fruit set in cotton (Table 2). However, their effectiveness differed regarding other quality parameters: fruits resulting from pollination by *T*. *fraterna* had significantly fewer non-intact seeds (LMER, *t*_59_ = −3.43, *P* = 0.002) than fruits after pollination by honeybees (LMER, *t*_150_ = 7.94, *P* = 0.013) and the other pollinator species. In comparison to fruits after pollination by the other wild bee and wasp species, seed weight and fibre weight of fruits significantly increased after pollination by honeybees (seed weight: LMER, *t*_150_ = 13.70, *P* = 0.004; fibre weight: LMER, *t*_150_ = 13.57, *P* = 0.005) and by *T*. *fraterna* (seed weight: LMER, *t*_59_ = 2.67, *P* = 0.008; fibre weight: LMER, *t*_59_ = 2.83, *P* = 0.005; Table 2, see Supplementary Table S2).

**Table 2:** Most frequent pollinator species of cotton in Burkina Faso and their effectiveness in terms of fruit set and quality parameters (n = 300 flowers; N = number of pollinator visits). *Apis mellifera* is a semi-wild honeybee species (i.e. some colonies are managed, while others are feral), all other species are wild bees. 4 species of wasps (Scoliidae) were observed as effective pollinators, the species were pooled due to very low number of visits. Wild bees with less than ten flower visits were pooled and analysed as remaining wild bees. Bold values refer to significant effects. Effect of field (random factor) was calculated using Chi square statistics.

Fixed effects	Fruit set		Non-intact seeds		Seed weight		Fibre weight		N
	*z*	*P*	*t*	*P*	*t*	*P*	*t*	*P*	
*Apis mellifera*	**2.48**	**0.013**	**7.94**	**0.013**	**13.70**	**0.004**	**13.57**	**0.005**	150
*Ceratina* spec.1	−0.23	0.820	−0.13	0.900	−1.01	0.312	−1.70	0.091	15
*Hypotrigona gribodoi*	0.07	0.948	−0.34	0.736	0.10	0.919	0.17	0.862	11
*Tetralonia fraterna*	−0.55	0.582	**−3.43**	**0.002**	**2.67**	**0.008**	**2.83**	**0.005**	59
23 remaining wild bees (pooled)	0.28	0.781	−1.57	0.133	0.02	0.984	−0.77	0.443	49
4 Scoliidae wasp species	1.80	0.073	−0.42	0.676	−0.80	0.423	−0.43	0.671	16
Random effect			***χ*** ^**2**^	***P***	***χ*** ^**2**^	***P***	***χ*** ^**2**^	***P***	
Field			0.577	0.4	11.5	<0.001	12.4	<0.001	

The level of significance is *P* < 0.05.

In sesame, honeybees were by far the most abundant (290 visits) and most effective pollinators in terms of seed- and fruit weight. The other bee pollinator species were infrequent visitors. Despite initiating fruit set, fruit and seed quality decreased. After pollination by the Mmason bee *Chalicodoma mephistolica* the number of non-intact seeds increased, and seed and fruit weight were decreased. Pollination by the stingless bee *Hypotrigona gribodoi* was not effective (see Supplementary Table S2).

### Pollinator dependency

**Figure 1:**
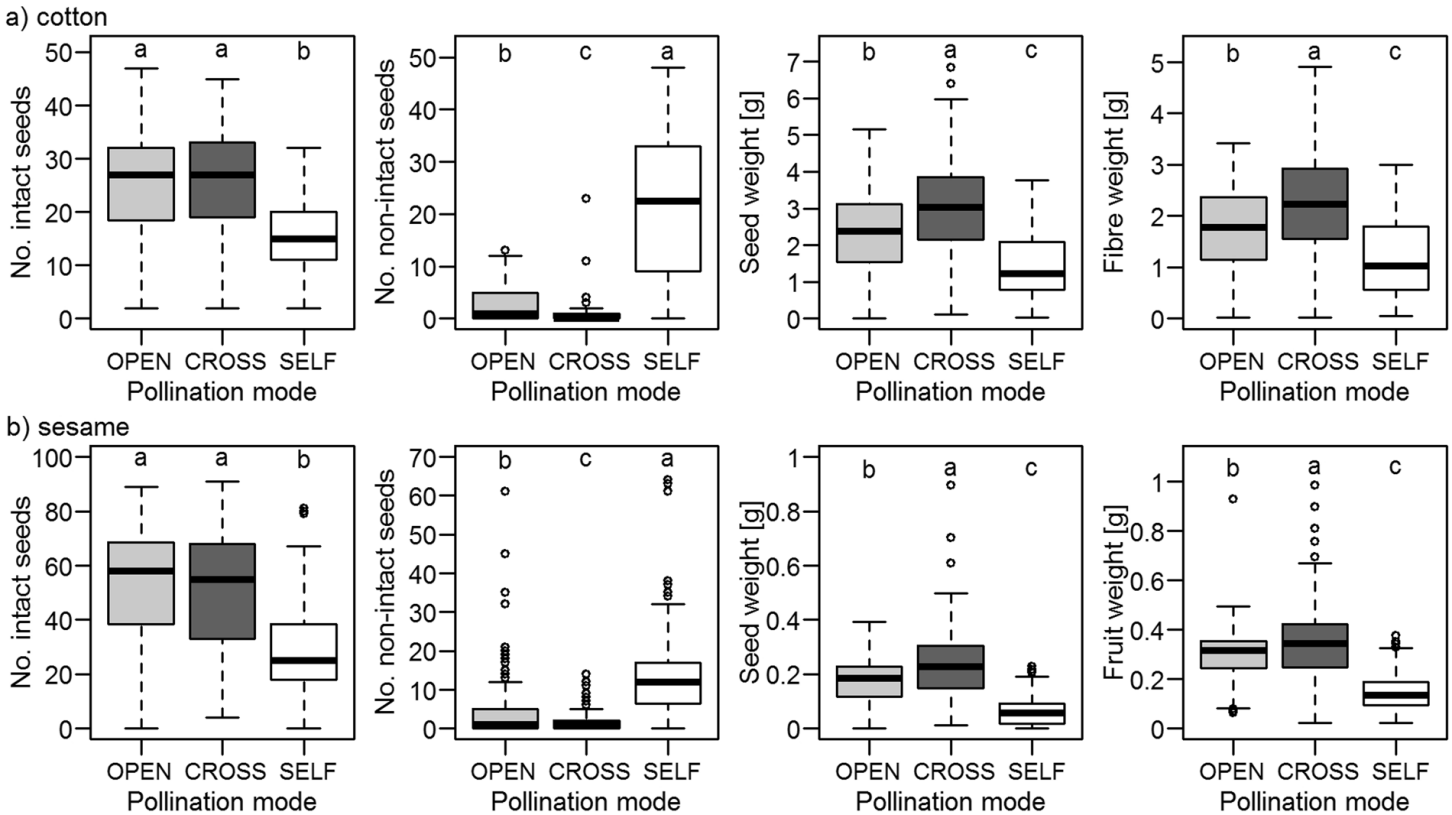
Fruit quality parameters of cotton (**a**) and sesame (**b**) resulting from pollinator dependency experiments in Burkina Faso, 2015. 550 flowers (2 flowers per treatment of 275 plants) from 11 fields per crop species were subjected to each pollination treatment: open natural pollination (OPEN, no manipulation), outcross-pollination (CROSS, emasculated flowers) and spontaneous self-pollination (SELF, exclusion of pollinators). Treatment effect was analysed using LMER with field and plant as random factor. Boxplots indicate the lower quartile, median and upper quartile, with whiskers extending to the most extreme data point that is no more than 1.5 times the interquartile range from the edge of the box. Letters indicate significance of differences in variable means at *P* ≤ 0.05.

Pollinator exclusion (spontaneous self-pollination, SELF) significantly decreased fruit set and fruit quality in both cotton and sesame. In cotton, natural pollination (OPEN) resulted in a mean fruit set of 48.73%, fruit set from self-pollination due to pollinator exclusion accounted for 37.45% (GLMER, *z* = −4.1, *P* < 0.001). In sesame, mean fruit set from natural pollination (OPEN) was 45.82%, in contrast to fruit set from self-pollination, which was 20.36% (GLMER, *z* = −9.3, *P* < 0.001). Similar results were found for all quality parameters (Fig. 1). In particular, the economically important parameter fibre weight in cotton (seed weight in sesame) significantly decreased by ca. 33% (ca. 59% in sesame) when pollinators were excluded (LMER, cotton: *t*_639_ = −8.8, *P* < 0.001; sesame: *t*_486_ = −9.8, *P* < 0.001). Fruits resulting from self-pollination contained eight times (cotton) or three times (sesame) as many non-intact seeds as fruits of the natural open pollination treatment (cotton: LMER, *t*_668_ = 26.9, *P* < 0.001; sesame: LMER, *t*_427_ = 11.4, *P* < 0.001). Outcross-pollination did not result in more abundant mean fruit set in both crops in comparison to natural pollination (cotton: 46.36%, sesame: 32.36%). However, cross-pollination by bees significantly increased fruit quality. In cotton, fruits resulting from outcross-pollination had significantly fewer non-intact seeds (LMER, *t*_641_ = −3.2, *P* < 0.01) and increased seed weight (LMER, *t*_613_ = 8.5, *P* < 0.001) and fibre weight (LMER, *t*_615_ = 7.1, *P* < 0.001) in comparison to the natural open pollination treatment (OPEN). In sesame, seed weight from outcross-pollination was ca. 41% higher than from mixed pollination of the control treatment (LMER, *t*_504_ = 8.6, *P* < 0.001; Fig. 1, see Supplementary Table S3).

### Economic valuation of bee pollination

The economic impact of bee pollination on producer profits was significantly beneficial in both crop species, and a loss of pollination service by bees would cause severe yield gaps.

The national average yield of cotton in Burkina Faso in 2015^47^ accounts for 1043 kg*ha^−1^. The mean yield of our study fields amounted to 953.91 kg*ha^−1^ (SD = 474.04 kg*ha^−1^; local cotton producer society Sofitex, unpublished data). This value corresponds to the control treatment. According to the national market prices of the Burkina Faso 2015–2016 cotton campaign, this translates into earnings of 364.39 USD per ha (SD = 181.08 USD per ha) were famers expenses of 140.87 USD per ha are still to be subtracted.

According to the data obtained in the pollinator experiments, an increase of yield quantity by bee-mediated outcrossing between 27% and 31% for cotton was constituted, which translates into additional earnings between 98.38 USD and 112.96 USD per ha.

On the other hand, the scenario of pollinator loss, and hence pure self-pollination of cotton flowers revealed a decrease of yield quantity between 33% and 43% This translates into a yield gap and net loss between 120.35 USD and 156.69 USD per ha. For sesame, the national average yield in Burkina Faso in 2015^47^ stands at 250–350 kg*ha^−1^. The mean sesame yield (seed weight) of our study fields amounts to 202.2 kg*ha^−1^ (SD = 44.26 kg*ha^−1^; local sesame producers, unpublished data). This value corresponds to the control treatment and equates to 86.95 USD per ha (SD = 19.03 USD per ha), were farmers expenses of 52.95 USD per ha are still to be subtracted.

Outcross-pollination by bees increased yield quantity between 37 and 42%, which translates into additional earnings between 32.17 USD and 36.52 USD per ha.

A lack of pollinators and their service would cause a decrease in yield quantity between 50 and 87% and would lead to a yield gap and net loss between 43.47 USD and 75,64 USD per ha.

## Discussion

The breeding system experiments have revealed that both investigated cotton and sesame varieties are partially self-compatible and facultatively xenogamous. They have a mixed mating system and produce fruits through self- and cross-pollination. The selfing rate is high in cotton and less pronounced in sesame. This is in line with other studies on cotton stating that cotton is mainly self-pollinating although cross-pollination occurs^20,23^. Cross-pollination is mostly attributed to insect pollination because cotton pollen is too large and heavy to be easily carried by air currents^53^. Studies on *Gossypium hirsutum* in Greece and California revealed less than 10% outcross pollination. They both state that cross-pollination of cotton highly depends on pollinator activity and is low in the absence of pollinators^54,55^. Our study revealed a relatively high rate of outcross-pollination which might be due to high pollinator abundances at our study sites. Furthermore, the cotton fields in our study had a size of about 1 ha and were embedded in a landscape mosaic of various other crop fields and savanna. In contrast, the studies in Greece and California were conducted on large holdings. Large monocultures may decrease pollinator abundance and hence lead to lower cross-pollination^56^. For sesame, our current finding contradicts a study stating that sesame is predominantly autogamous^57^, but is in line with another study revealing best performance in sesame in Brazil after cross-pollination^58^. Nevertheless, crossing rates reported in some studies ranged from less than 10% to 68%^59,60^. The outcrossing rate of the sesame variety used in this study was 32.36%. Such high variations in levels of pollinator dependence for outcross pollination is typical of crop species with mixed mating systems^19^ and evidencing the need for breeding system studies and pollinator dependence experiments for each variety and environmental setting.

Despite high selfing rates, self-pollination led to clear signs of inbreeding depression, being highest in both species for number of seeds, and additionally, in cotton, for fruit set and in sesame for seed germination.

We recorded honeybees and a plethora of wild bees as pollinators of cotton and sesame at our study sites, with honeybees and the long-horned wild bee *Tetralonia fraterna* being most effective in terms of fruit set and fruit quality. Since flower visitor abundances were very uneven, one might argue that abundance might account for pollinator efficiency. This might be true for the two most efficient species recorded. In a previous study based on pantraps *Apis mellifera* (364 specimens in cotton fields, 142 in sesame) and *Tetralonia fraterna* (120 specimens in cotton fields) belonged to the top three most abundant bee species in cotton fields assessed in our study area within two sampling years (2014 and 2015) (KStein, data unpublished). The pantrap study revealed that the stingless bee *Hypotrigona gribodoi* was most abundant in both cotton (4253 specimens) and sesame (165 specimens) fields. In the present study though, this species turned out to be less efficient, indicating that sole abundance does not stand for species efficiency. It might rather be the pollen collecting behaviour and body size that account for bee species efficiency as pollinators. Pantraps do not allow for concluding on a species to be a pollinator, since the insects might have been caught by accident and do not necessarily need to be pollinators. This method can only be used to assess the community of “potential pollinators”. Our finding underlines the importance of direct pollinator observations on the flowers and an assessment of their pollination success as we did with the pollinator efficiency approach.

Many native bee species other than honeybees have been shown to visit crops^61^ and have been recognized for their role in increasing and stabilizing crop-pollination services^62,63^. Bees, and in particular wild bees, are known to improve seed set, quality, and commercial value of a variety of crops^29,64^. A meta-study including 90 studies from five continents found that wild bee communities contributed about the same to crop production as managed honeybees did^65^. Hence, wild bees complement, in a number of ways, the service provided by honeybees; first biologically, by enhancing the efficacy of honeybee pollination in some cases^6^, and then economically, by insuring against pollination shortages. Availability of accurate estimates of this value could improve land use planning by quantifying the costs and benefits of conserving habitats for pollinators in agricultural landscapes.

Our research revealed that cross-pollination by honeybees and wild bees significantly increased yield quality in both cotton and sesame, which is in line with the findings of other studies on cotton^23^ and on sesame^58^. However, these studies were carried out in Australia and Brazil and our results reveal even higher benefits from bee pollination in terms of fruit quality. To our knowledge, our study is the first from the sub-Saharan West African country of Burkina Faso. Quality significantly increased for the economically important parameters of fibre (lint) and seed weight by two thirds in cotton, and seed weight was tripled in sesame. A higher number of intact seeds and increased seed weight per fruit are desirable features for cotton and sesame from both the commercial and ecological point of view, which is an important indicator of plant reproductive success. A loss of bee pollinators concomitant with self-pollination of the crop species would result in yield gaps between 33% and 43% for cotton and 50% and 87% for sesame, respectively. Furthermore, seeds from outcross-pollination were more viable and vital, and germinated more readily, contrary to seeds from self-pollination. This finding is relevant for the smallholders and cotton companies, since they use the seeds from the previous harvest for next year’s sowing season. A high portion of outcrossed seeds significantly enhances the success of next year’s cultivation. Thus, our study clearly reveals the value of bee pollination to cotton and sesame production in smallholder systems in sub-Saharan Africa. Since it lacks detailed social data on the livelihood of the farmers, the economic evaluation in this study only allows broad conclusions on potentially increased earnings or losses from existing or lacking pollination services in relation to national statistics.

In the context of human population growth and land conversion with natural habitat loss as one of the main drivers of bee decline^66,67^, and increasing climate unpredictability^68^ especially for West Africa, targeted establishment and preservation of semi-natural habitats would consistently increase the diversity and abundance of wild pollinators^62^. The expected agricultural loss in the absence of animal pollination is estimated at 5–7.5% in Burkina Faso^56^. As with a number of previous studies, estimates of the economic benefits of pollination services are limited by the assumption of constant market prices^69^. In particular, the benefits reported here may vary depending on the presence of other inputs such as fertilizers and pesticides or ecosystem services^70^. For our calculation, we have used the market prices of 2^nd^ class quality cotton and the lowest market price of sesame seeds.

Additionally, world cotton production is projected to grow annually by 2.1% over the next ten years (2.3% in West Africa). Besides Brazil, least developed countries in sub-Saharan Africa are also expected to augment their cotton exports, increasing their share of world trade growing from 8 to 9% by 2024. Cotton consumption is limited throughout sub-Saharan Africa, and many countries export virtually their entire production^71^. In sesame, the annual production growth rate in Burkina Faso is estimated to be 18.4%^15^. Based on a sesame seeds export quantity of 58,650 tons in 2012, which equates to an export value of 56,730^1000 USD^15^, the loss of pollination service would lead to a yield loss of 34,498 tons on a national scale and would have severe economic impact on the country.

## Conclusion

By combining results from exclusion experiments, pollinator surveys and field manipulations, this study for the first time quantifies the contribution of bee pollinators to smallholders’ cash crop production in Burkina Faso. Measures of pollinator diversity and effectiveness were combined and compared between species to quantify their contribution to cash crop production and economic output in West Africa. This study is also unique in that pollinator species diversity and efficiency were assessed, and that an in-depth assessment was provided, tracking multiple steps of the different modes of pollination from the breeding system to the F1-generation.

As our knowledge on pollination efficiency of different pollinators to different crops grows and consolidates globally^72^, the concepts used in this study can be applied to better quantify economic impacts of different components of the pollinator community on crop production. Ultimately, this can result in more holistic models of pollination service provision and better forecasting of the risks of pollinator declines^68,69^.

These findings have implications for the management of insect pollination services in cash crop fields such as cotton and sesame, and highlight potential consequences of any decline in specific taxa, thereby advocating the conservation of bee populations.

## Electronic supplementary material

Supplementary Information
