# Supplementary material for: Bee pollination increases yield quantity and quality of cash crops in Burkina Faso, West Africa
Source: Sci Rep. 2017 Dec 18;7:17691. doi: 10.1038/s41598-017-17970-2 (PMC5735132; doi:10.1038/s41598-017-17970-2)
Supplement: Supplementary file 1 — Supplementary Information [file 41598_2017_17970_MOESM1_ESM.pdf]

Bee pollination increases yield quantity and quality of cash crops in Burkina Faso, West Africa

Katharina Stein[1,4*], Drissa Coulibaly[2], Kathrin Stenchly[3], Dethardt Goetze[4], Stefan Porembski[4], André Lindner[5], Souleymane Konaté[2] and K. Eduard Linsenmair[1]

[1]Biocenter, Department of Animal Ecology and Tropical Biology, University of Wuerzburg, Wuerzburg, Germany; katharina.stein@uni-wuerzburg.de; ke_lins@biozentrum.uni-wuerzburg.de

[2]Unité de Formation et de Recherche des Sciences de la Nature, Unité de Recherche en Ecologie et Biodiversité, Université Nangui Abrogoua Abidjan, Côte d'Ivoire; coulibalydriss2008@yahoo.fr; konatesoul_sn@una.edu.ci

[3]Faculty of Organic Agricultural Sciences, Universität Kassel, Germany; stenchly@uni-kassel.de

[4]Institute of Biological Sciences, Department of Botany and Botanical Garden, University of Rostock, Rostock, Germany; dethardt.goetze@uni-rostock.de; stefan.porembski@uni-rostock.de; katharina.stein@uni-rostock.de

[5]Centre for International Postgraduate Studies of Environmental Management – CIPSEM, Technische Universität Dresden, Dresden, Germany; andre.lindner@tu-dresden.de

Supporting information

Table S1. Pollination treatments to determine the breeding systems of conventional upland cotton (*Gossypium hirsutum*) and sesame (*Sesamum indicum*). Treatments were implemented on 100 flowers (each 2 flowers per treatment per 50 plants) per crop species on 1 field each. For the pollinator dependence experiments, test no. 1 (OPEN), 2 (SELF) and 5 (CROSS) were conducted on 11 fields per crop species (n = 550 flowers/test). According to Dafni (1992), modified.

**Test**	**Treatment**			**Remarks**
1 control (C) , OPEN	unbagged	untreated	free pollination	evaluation of pollination rate under natural conditions
2 spontaneous self-pollination (SS), SELF	bagged	untreated	pollen source: same flower	measuring need for pollinators
3 hand self-pollination (HS)	bagged	emasculated	pollen source: different flower of same plant	indication of self-(in)compatibility systems
4 hand cross-pollination (HC)	bagged	emasculated	pollen source: min.10 other individuals of genet	indication (with no. 3) of self-(in)compatibility
5 cross-pollination under natural conditions (OC), CROSS	unbagged	emasculated	free pollination	evaluation of selfing-rate with no.1 and 2

Table S2. Most abundant pollinator species of cotton and sesame and their effectiveness in terms of fruit set and quality parameters (n = 300 flowers/crop). Values indicate mean ± SD. Only pollinator species that initiated fruit set are listed. *Apis mellifera* is a partly managed honeybee species, all other named bees are wild bees. Very low number of visits were recorded for the remaining seventeen wild bee species and four wasp species (Scoliidae) not named individually. They were grouped.

		Reproductive parameters				
Pollinator species	n visits	fruit set [%]	n intact seeds	n non-intact seeds	seed weight [g]	fibre weight [g]
**cotton**						
Apis mellifera	150	63.33 ± 48.35	30.21 ± 05.02	2.87 ± 2.48	3.73 ± 0.65	2.96 ± 0.52
Tetralonia fraterna	59	54.24 ± 50.25	31.69 ± 05.28	0.69 ± 0.82	4.15 ± 0.98	3.35 ± 0.82
Ceratina spec.1	15	60.00 ± 50.71	19.00 ± 14.20	1.49 ± 2.23	2.09 ± 1.55	1.62 ± 1.19
Hypotrigona gribodoi	11	62.50 ± 49.45	15.91 ± 15.63	1.04 ± 2.01	2.06 ± 1.83	1.62 ± 1.45
other 17 wild bee species	66	75.61 ± 43.48	20.07 ± 14.38	1.39 ± 1.73	2.39 ± 1.59	1.81 ± 1.19
Scoliidae - 4 wasp species	16	87.50 ± 34.16	26.50 ± 06.58	2.36 ± 3.61	2.88 ± 0.82	2.37 ± 0.46
**sesame**						
Apis mellifera	290	46.55 ± 49.97	50.99 ± 19.62	2.10 ± 07.25	0.20 ± 0.07	
Chalicodoma mephistolica	5	20.00 ± 44.72	0	46 ± 20.57	0	
Hypotrigona gribodoi	2	50.00 ± 70.71	0	0	0	

Table S3. Fruit set and quality parameters of cotton and sesame resulting from pollinator exclusion experiments. Each 550 flowers from 11 fields per crop species (conventional cotton : *Gossypium hirsutum* ; sesame : *Sesamum indicum*) were subject to the pollination treatments control (no manipulation, OPEN natural pollination), outcross-pollination (emasculation of flowers, CROSS) and spontaneous self-pollination (exclusion of pollinators, SELF). Values indicate mean ± SD.

Reproductive Parameters	Treatments		
	OPEN	CROSS	SELF
**cotton**			
fruit set [%]	48.73 ± 50.02	46.36 ± 49.91	37.45 ± 0.48
n intact seeds	25.23 ± 9.73	25.90 ± 9.33	15.87 ± 6.69
n non-intact seeds	2.69 ± 3.19	0.59 ± 1.74	21.07 ± 13.35
seed weight [g]	2.35 ± 1.11	3.03 ± 1.32	1.42 ± 0.86
fibre weight [g]	1.73 ± 0.81	2.22 ± 1.01	1.15 ± 0.71
germination [%]	61.35 ± 26.78	72.16 ± 23.94	47.09 ± 28.66
**sesame**			
fruit set [%]	42.00 ± 49.39	29.50 ± 45.64	18.56 ± 38.91
n intact seeds	51.06 ± 23.83	51.44 ± 21.35	28.16 ± 20.61
n non-intact seeds	4.10 ± 7.33	1.73 ± 2.49	13.71 ± 12.07
seed weight [g]	0.17 ± 0.09	0.24 ± 0.13	0.07 ± 0.06
fruit weight [g]	0.30 ± 0.10	0.34 ± 0.15	0.15 ± 0.07
germination [%]	26.29 ± 23.17	62.16 ± 37.14	0.56 ± 10.11
